# New Partner, New Order? Multipartnered Fertility and Birth Order Effects on Educational Achievement

**DOI:** 10.1007/s13524-020-00905-4

**Published:** 2020-09-15

**Authors:** Mats Lillehagen, Martin Arstad Isungset

**Affiliations:** Department of Sociology and Human Geography, University of Oslo, PO Box 1096 Blindern, 0317 Oslo, Norway

**Keywords:** Birth order, Siblings, Education, Multipartner fertility

## Abstract

A substantial amount of research shows that younger siblings perform worse than their older sisters and brothers in several socioeconomic outcomes, including educational achievement. Most of these studies examined stable families and excluded half-siblings. However, the increasing prevalence of multipartnered fertility implies that many children grow up in nonnuclear families. We examine whether there is evidence for birth order effects in this context, which offers an opportunity to test and potentially expand the explanatory scope of the two main theories on birth order effects. We use comprehensive Norwegian registry data to study siblings in the 1985–1998 cohorts born to mothers or fathers who parented children with at least two partners. We provide evidence for negative effects of birth order on lower secondary school grades in both cases. Children born to fathers displaying multipartnered fertility tend to have lower grades than older full siblings but perform more similarly or better compared with older half-siblings. For siblings born to mothers with the multipartnered fertility pattern, later-born siblings do worse in school compared with all older siblings. This indicates that negative birth order effects tend to operate either within or across sets of full siblings, depending on the sex of the parent displaying multipartnered fertility. We argue that these findings can be explained by a combination of resource dilution/confluence theory and sex differences in residential arrangements following union dissolutions. We also suggest an alternative interpretation: maternal resources could be more important for generating negative birth order effects.

**Electronic supplementary material:**

The online version of this article (10.1007/s13524-020-00905-4) contains supplementary material, which is available to authorized users.

## Introduction

Being later-born confers a negative effect on several outcomes, including educational performance and choices (Barclay et al. 2017; Black et al. 2005; De Haan 2010; Kristensen and Bjerkedal 2010), intelligence scores (Black et al. 2011; Boomsma et al. 2008; Kristensen and Bjerkedal 2007; Sulloway 2007), income and choice of profession (Björklund et al. 2004; Grinberg 2015; Kantarevic and Mechoulan 2006), as well as health and mortality (Barclay and Myrskylä 2014; Black et al. 2016).

The extant literature, although both methodically rigorous and empirically rich, generally limits its scope to studying stable nuclear families. This approach has clearly served as a good starting point for birth order research given that traditional family patterns are still the most prevalent in Western countries, including Norway (Lappegård and Rønsen 2013; Thomson 2014). However, an increasing proportion of children and adults find themselves in families characterized by greater levels of instability and complexity (Andersson 2004; Andersson and Kolk 2015; Cancian et al. 2011; Kalmijn 2015a). Half-siblings, stepsiblings, stepparents, and a larger number of less clear-cut family relations have become more common (Guzzo 2014; Lichter et al. 2010; Thomson 2014).

An important part of this picture is the increasing prevalence of multipartnered fertility—that is, parenting children with more than one partner during one’s reproductive history (Carlson and Furstenberg 2006; Lappegård and Rønsen 2013; Thomson et al. 2014). For example, estimates for representative samples or populations from the United States, Australia, Sweden, and Norway found that between 10% and 20% of mothers and fathers display this fertility behavior based on data of births occurring from the 1980s to the early 2000s (Thomson et al. 2014). In this study, we extend the existing literature on birth order effects by considering such complex fertility histories. We utilize comprehensive Norwegian registry data and study the association between birth order and educational achievement. Our main samples are restricted to siblings born to either fathers or mothers who have parented at least three children in total with more than one co-parent. We analyze two samples that include siblings who share either a father or a mother displaying multipartnered fertility.

We examine three research questions. First, we ask whether there are negative birth order effects on educational outcomes, measured by average grades at the end of compulsory education, in the context of complex fertility patterns. The answer is not obvious: older siblings are more likely to be affected by adverse consequences of union dissolution and other related factors (Bronte-Tinkew et al. 2009; Fomby and Osborne 2017; Sigle-Rushton et al. 2014; Tach et al. 2010) that could attenuate, cancel out, or even reverse negative birth order effects.

Second, we investigate whether birth order effects are associated with the birth order of the child within the same *set*—that is, among full siblings who share both parents, with the birth order of the child relative to all full *and* half-siblings, or both. This distinction, which becomes relevant when considering multipartnered fertility, allows us to answer questions such as whether being the overall firstborn to a parent displaying this fertility pattern is better in terms of school achievement compared with being the firstborn within a set of full siblings. More generally, we are able to ask whether birth order effects operate mainly *within* or *across* sets of full siblings.

Third, we ask whether these associations differ depending on the sex of the parent displaying multipartnered fertility. We expect such differences because residential arrangements and the frequency of parental contact tend to vary with sex for parents who display multipartnered fertility.

In a broader societal perspective, this study provides a better understanding of the intrafamily distribution of socioeconomic advantages and disadvantages associated with an increasingly frequent type of fertility behavior. Norway is a good starting point for such analyses given that it is often considered to be among the forerunners of the second demographic transition (Lappegård et al. 2011; Lesthaeghe 2010).

## Theory and Previous Research

We begin by presenting the two dominant theories of birth order. Both theories emphasize the importance of children living together or at least interacting frequently with both parents, competing for resources, tutoring each other, and so forth (Black et al. 2005; Hertwig et al. 2002).^1^

### Resource Dilution Theory

The main idea behind the resource dilution theory is that the firstborn child has the exclusive attention and resources of the parents, whereas such resources are diluted for younger siblings who must share them with their older siblings (Downey 2001). An underlying assumption is that parents attempt to distribute their finite resources equally between the children. Given that early investments are more important (e.g., Carneiro et al. 2015), this leads to negative effects of birth order on socioeconomic outcomes. The theory has received much attention, and studies have tested it using detailed data on actual time spent with children (Pavan 2016; Price 2008), and quality time more specifically (Monfardini and See 2016; Price 2008). The importance of smoking during pregnancy (Lundberg et al. 2010), use of child health care services (Butikofer et al. 2015), and breastfeeding (Buckles and Kolka 2014) have also been investigated. Although the theory is generally well supported empirically, the questions of which type of resources matter for birth order effects and their relative importance remain contested (e.g., Monfardini and See 2016).

### Confluence Theory

Another important and closely related theory is the confluence theory (Conley 2004; Zajonc 1983; Zajonc and Markus 1975). The central explanatory concept in this theory is referred to as the *average intellectual climate* of the family. With each additional child born into the family, the intellectual climate drops not only for the new child but also for the earlier-born. The intellectual family environment is therefore better for the firstborn than the second-born, for instance: the firstborn benefitted from initially having the intellectual environment exclusively with his/her parents. The confluence theory also postulates a positive effect for older children tutoring their younger siblings (e.g., Silles 2010), which yields negative birth order effects. Although the empirical expectations associated with this theory are generally similar to those of resource-based theories, some of the predictions made are more specific. The spacing between births should play an important role in generating differences in outcomes by birth order, and it has been claimed that the negative effect of being born later will reverse at the age of 11 (Zajonc et al. 1979). Thus far, no decisive evidence exists, and some of these more specific predictions have not been supported in recent studies (Barclay and Kolk 2018; Bonesrønning and Massih 2011; Härkönen 2014; Lehmann et al. 2018).

### The Two Theories and Multipartnered Fertility

The two theories suggest that regular interaction with both parents in a common family environment is the most important precondition for the existence of negative birth order effects. In most previous studies, excluding children with half-siblings made such interactions between siblings and both parents very likely to occur in the samples studied. Many (if not most) of the children in our samples, however, experienced union dissolutions. In some cases, they never lived with both parents simultaneously. Still, we argue that it is reasonable to expect negative birth order effects based on the two theories, but three key issues need to be accounted for to derive more specific predictions: (1) the distinction between overall and full biological birth order, (2) the significance of union dissolutions and family complexity, and (3) sex-differentiated parental behavior (discussed in the upcoming section on different expectations for the two samples). We will now discuss these in turn beginning with the first, which is crucial for evaluating the significance of the latter two.

#### *Overall and Full Biological Birth Order*

In studies of nuclear families, birth order is assigned based on the year of birth for all siblings who share the same mother *and* father. When considering birth order in the context of multipartnered fertility, however, there are multiple fathers and/or mothers involved. We distinguish between two types of birth order to provide an adequate descriptive overview and because the implications derived from the two theories discussed will differ depending on the type of birth order.

First, we define the *overall* birth order based on the year of birth for each child relative to all full and half-siblings with whom one shares the same father or mother—that is, all children from both *sets*. Second, we refer to the *full biological* birth order of each child based on a child’s year of birth relative to other full siblings parented by the same mother *and* father—siblings *within* the same set. The latter definition of birth order is similar to those used in previous studies, whereas the first is novel.

An example will make this important distinction more transparent. Consider our first sample, which includes siblings born to mothers who have parented children with more than one partner. Say that a mother has four biological children: two with the first father and two with the second. These four children are assigned an overall birth order from 1 to 4 based on their year of birth in ascending order. For the full biological birth order, however*,* the two oldest children in this example are assigned values of 1 and 2 based on their year of birth relative to each other. The last two born are also given the values of 1 and 2 because they are (respectively) first- and second-born in the mother’s last set. In this example, the values for the two types of birth order are identical for the two oldest children (overall = 1, 2; full biological = 1, 2) but different for the two youngest (overall = 3, 4; full biological = 1, 2).

#### *Union Dissolution and Family Complexity*

In a nuclear family setting, parental fixed effects make it possible to provide a robust *ceteris paribus* estimate of birth order effects. In the context of multipartnered fertility, having a higher birth order is correlated with a lower probability of having experienced a divorce as well as a higher probability of having an older half-sibling. Further, children in the second set are more likely to experience greater family stability, which could lead to confounding of negative effects of *both* full biological and overall birth order on educational achievement.

One potential complication is related to the fact that children of the first set in both our samples have experienced a union dissolution at some point, typically before the first child/children of the second set is/are born. Union dissolutions affect children’s educational achievements negatively (e.g., Steele et al. 2009). The occurrence of union dissolutions could affect birth order effects within each set (i.e., the full biological birth order effects) if this event affects children differently depending on their age (Sigle-Rushton et al. 2014). Previous studies taking both birth order and union dissolutions into account simultaneously for siblings who share both parents have found that this is generally not the case (Kantarevic and Mechoulan 2006; Kristensen and Bjerkedal 2010; Sigle-Rushton et al. 2014). Therefore, we do not expect union dissolutions to affect the magnitude of any full biological birth order effects on educational attainment.

Still, union dissolution and family complexity or instability may cause differences *between* sets of siblings, which could affect our estimate of the *overall* birth order effects. Union dissolutions are likely to affect children in the first set negatively given that these children will have experienced this event in almost all cases. Additionally, many researchers writing about multipartnered fertility and complex families have been concerned about the more general disadvantages associated with growing up in these types of situations, including worse educational outcomes (Bernardi and Radl 2014; Bronte-Tinkew et al. 2009; Fomby and Osborne 2017; Fomby et al. 2016; McLanahan et al. 2013; Tach et al. 2010). A common argument is that the complexity of such arrangements leads to considerable stress for both children and parents (Petren 2017). We argue that these negative effects are likely to work in the same direction as union dissolutions, affecting children of the first set negatively, because we expect the second union to provide a more stable environment (Beaujouan 2016; Poortman and Lyngstad 2007).

These factors therefore imply a positive correlation between school achievement and overall birth order and thus an association opposite of the typically negative birth order effect. This implies that the estimated magnitude of the overall birth order effect in this context will be biased downward (become less negative), and what we obtain is therefore an estimate of this effect net of these offsetting mechanisms.

### Different Expectations for the Two Samples in Light of the Resource Dilution and Confluence Theories

In both the resource dilution and the confluence theory, parent-child interaction causes birth order effects. In our samples, union dissolutions have occurred, and the parent-child interaction is not the same as in nuclear families. Mothers and fathers will typically create a family environment together with their child(ren) as a couple before they split up and the child(ren) start to live with their mother, while the father may or may not still be involved. We expect this to have different implications for birth order effects in our two samples.

For children to *fathers* who display multipartnered fertility, siblings in each set typically grow up in parental environments that are separate most of the time. Although all children will typically spend some time with both parents, the children of the first set (in particular) will typically spend much of their upbringing with their mother and their full siblings (and occasionally stepfathers). From the point of view of the two theories, this implies a discontinuity compared with the nuclear family context. Initially, both parents are present, which resembles the nuclear family mechanism. In the father-based sample, we expect that this will mainly give rise to birth order effects between full siblings (i.e., full biological birth order effects). Although the father is present and contributes (both before and after the union dissolution) to some degree, his presence is not consistent in the same way as that of the children’s mother, with whom they typically reside. In other words, because a father displaying multipartnered fertility spends less time distributing his resources between *all* his children simultaneously (because his pattern of investment varies across sets with time), we are less likely to see evidence for overall birth order effects in the father-based sample. However, the combined efforts of the father and the mother within each set will give rise to full biological birth order effects.

We have different expectations for the *mother* sample, based on the same premises. In this situation, both full and half-siblings will spend more time interacting in a common environment. Although it clearly varies across time whether both the father and mother (or stepparents) are present, the mother’s continued presence provides a consistency that differs from that seen in the father-based sample. Importantly, this implies that the mechanisms specified in the two theories can operate for all full and half-siblings that share the same mother. In other words, although both mothers and fathers contribute in both samples, the residential situations allow for continuity across sets in this case that was not present in the other sample, therefore giving rise to *overall* birth order effects. We therefore expect birth order mechanisms to operate mainly *within* sets of full siblings in the father-based sample and *across* in the mother-based sample.

This argument is based on some important premises, which are supported in existing literature. Previous evidence from Norway for the period covered by our data (children in the 1985–1998 birth cohorts) indicates that mothers tend to live with all or most of their biological children in the overwhelming majority of cases in which the parents have separated or never lived together (Jensen and Clausen 1997; Lappegård and Rønsen 2013). In the far–less common case of children registered as living with their fathers, some type of joint physical custody is present more often than not, implying that the mother is typically consistently involved with all her children in this situation as well (Kitterød et al. 2016). Furthermore, in Norway during the period studied, full paternal physical custody was almost non-existent, and joint physical custody was extremely rare when children were registered as living with their mothers. Jensen and Clausen (1997) documented a prevalence of approximately 4% of cases at the end of our study period. This was also the general finding in studies based on data from many other Western countries in this period (e.g., Baxter 2012; Cancian and Meyer 1998; Singer 2008; Sodermans et al. 2013; Tach et al. 2010). Also, although fathers are often still involved without physical custody, both Norwegian and international evidence indicate that parenting children with new partners is associated with less paternal involvement with older children born to a previous co-parent (Berger et al. 2012; Kalmijn 2015b; Kitterød and Lyngstad 2012; Manning and Smock 1999, Skjørten et al. 2007).

Our expectations are summarized in Table 1.

**Table 1:** Empirical expectations for each sample and birth order types based on the theoretical discussion

Sample	Birth Order Type	
	Full Biological	Overall
Father	Yes	Weak/unclear
Mother	Yes	Yes

## Data and Sample Restriction

We use comprehensive registry data that cover the complete population of Norway. We link information on individuals from several registries through a nonidentifiable and unique ID number and study the relationship between birth order and grades (*Grunnskolepoeng*, hereafter referred to as *school points*) at the end of compulsory education (lower secondary school). The population registry enables us to link all individuals to their parents and thereby to their full siblings and half-siblings. Information on school points was obtained from a registry of educational information, which contains data on nearly all pupils during the period under observation.

Our samples include all children of mothers (the mother-based sample) or fathers (the father-based sample) who parented at least three children in total with at least two partners. One or more of the co-parent(s) in each sample could also display multipartnered fertility. To estimate the full biological birth order, the same person must be the co-parent of at least two of these children. Information on lower secondary school points are measured at age 16 and are available from 2001 to 2014. This leaves us with information on the cohorts from 1985 to 1998. We therefore include children of fathers or mothers whose first child was born in or after 1985 and who have at least two siblings, which implies that we have information on school points for all older full and half-siblings for everyone in our sample.

Individuals with younger siblings who are not old enough to have finished lower secondary school in 2014 are included in the main samples. We exclude all siblings of children with missing information on school points. Fifth-born and later-born children were also excluded because of the very small number of observations in these categories. Neither exclusion affected our main findings.

Our main analyses use a within-father/within-mother design, which creates additional data demands. Because the fixed-effects estimations require variation in birth order between children of the same father, the same mother, and combinations of fathers and mothers, we exclude children of plural births. These restrictions leave us with 20,776 and 19,508 observations in the father-based and mother-based samples, respectively. Additionally, we include results from a nuclear family sample that uses the same criteria but excludes those with half-siblings, and which is limited to parents with four or fewer children (*N* = 396,864).^2^ This sample therefore includes only those children of two parents who exclusively parented children with each other.

## Statistical Approach

### Explanatory Variables: Birth Order

This study uses a distinction in two types of birth order. First, we define the overall birth order based on the year of birth for each individual relative to all full and half-siblings with the same father (father sample) or mother (mother sample). We assign a value between 1 and 4 to each child. Overall birth order is included in our models as a set of four dummy variables for those who are firstborn (reference group), second-born, third-born, and fourth-born.

The second type of birth order is full biological birth order of each child, similar to typical measures of birth order used in within-family studies. We assign a value to the child based on his or her year of birth relative to other full siblings parented by the same mother *and* father. The measure is also included as a set of dummy variables with firstborn as the reference group, and we also include dummy variables for second-born and third-born.

### Outcome Variable

#### *School Points at the End of Compulsory Education*

We measure educational success as the school point average at the completion of lower secondary school, which occurs at age 16. Lower secondary school comprises grades 8–10 and is mandatory in Norway. The school point variable combines the most important grades achieved throughout this level of schooling. Grades are awarded on a scale of 1 to 6, and both overall achievement grades and exam grades are included. The averages of all grades are calculated and then multiplied by 10 to obtain the value of the variable on a metric scale. The grading system was altered slightly in 2007, and a tendency for the average to rise somewhat during the period has been documented (Bakken and Elstad 2012). The inclusion of control variables for birth cohort enables us to adjust for such effects.

### Covariates

In addition to our measures of birth order, we include a set of individual-level covariates, which have been shown to be important for the proper estimation of birth order effects and are therefore considered standard (see, e.g., Black et al. 2005; De Haan 2010; Härkönen 2014; Kristensen and Bjerkedal 2010).

#### *Parents’ Age at Birth*

Subtracting the parent’s year of birth from each child’s year of birth gives us the parent’s age at birth. We include this information as two sets of dummy variables with six categories (16–19 years old, 20–24 years old (the reference category), 25–29 years old, 30–34 years old, 35–39 years old, and 40 years or older).

#### *Child’s Year of Birth*

Child’s year of birth is included as a set of dummy variables for each year from 1985 to 1998, with the first year as the reference category. This variable serves as a standardizer of the grade scale as well as a cohort control.

#### *Child’s Gender*

Because gender is known to be important to educational outcomes, the child’s gender is included as a dummy variable to standardize the coefficients (0 = male, 1 = female).

#### *Variables Included in Additional Analyses*

We include measures for spacing (full samples) and cohabitation (available for children born from 1987 to 1998) in several additional analyses in the online appendix. We provide more details on these variables in the relevant tables.

### Analytical Strategy

We estimate three main models with the grade average at the end of compulsory education as the outcome variable in all three. We use linear regression models estimated separately for each sample. Our approach enables us to apply father or mother fixed effects (depending on the sample), and we include control variables for the child’s gender, year of birth, and the age at birth of both parents. We examine the relationship between school points and the overall birth order (Model 1) and the full biological birth order (Model 2). Model 3 is similar to the first two but includes *both* types of birth order, which allows us to examine the relative importance of each type given that the other is adjusted for. This strategy is possible because the two types of birth order have different values for some children in our sample, as indicated in the examples discussed earlier in connection with the definition of the birth order measures. We use a similar specification when we estimate birth order effects for the nuclear family sample, but the clustering in the fixed effects in this model are based on combinations of mothers’ and fathers’ IDs.

#### *The Educational Attainment of the Co-parent*

In most rigorous studies of birth order, effects are estimated based on a within-family design—that is, using fixed effects for combinations of mothers and fathers. This estimation strategy, however, is not applicable in all cases given our main samples and analytical approach because every mother or father is required to have parented children with at least two co-parents. It is possible that these co-parents differ systematically in characteristics that affect the educational success of their children through social or genetic transmission. This could potentially cause or confound birth order effects associated with the *overall* birth order in cases where half siblings are compared. To consider this issue, we first include a measure of the educational attainment of each co-parent as a covariate. This makes almost no difference to our estimates (see Tables A6 and A7 in the online appendix), and because the inclusion of this mediating variable could introduce endogeneity issues, we opt to not include it in our preferred models (Morgan and Winship 2015).

## Results

Table 2 provides descriptive information about the most important variables included in our analyses. The descriptive results are quite similar for the two samples. However, fathers displaying multipartnered fertility parent slightly more children overall with more partners, as reflected in the slightly higher proportions in the upper birth order categories.

**Table 2:** Descriptive statistics for the father-based and mother-based samples

	Father-Based Sample		Mother-Based Sample	
Variables	*N*/Mean	SD/%	*N*/Mean	SD/%
Outcome Variable				
Grade average (mean, SD)	39.00	8.9	37.96	8.87
Birth Order (*N*, %)				
Overall birth order				
1	6,476	31.17	6,152	31.53
2	6,476	31.17	6,152	31.53
3	6,476	31.17	6,152	31.53
4	1,357	6.53	1,053	5.40
Full biological birth order				
1	13,171	63.39	12,444	63.79
2	6,872	33.07	6,502	33.33
3	736	3.54	563	2.89
Combinations of Number of Children and Partners (*N*, %)				
1-2	9,576	46.08	9,276	47.55
2-1	5,736	27.60	6,339	32.49
1-3	1,990	9.58	1,173	6.01
2-2	1,452	6.99	1,176	6.03
3-1	842	4.05	737	3.78
Other	1,183	5.70	808	4.14
Control Variables (mean, SD)				
Year of birth	1991.8	(4.00)	1991.8	(4.07)
Mother’s age at birth	26.02	(4.98)	26.04	(4.93)
Father’s age at birth	29.14	(5.44)	29.21	(6.04)
Number of Observations	20,776		19,508	

*Source:* The authors’ own calculations based on registry data

Because of the way our main samples were constructed, they include equal numbers of first-, second-, and third-born siblings when the *overall* birth order is considered. We also include information on the number of children per partner for the most common combinations to provide a better overview of our sample: 1-2 indicates one child with the first partner and two with the second, 2-1 indicates two children with the first partner and one with the second, and so on. These combinations are fairly equally distributed between the samples, although the 1-3 combination (one child with the first partner and three with the second) is slightly more common among fathers.

**Fig. 1:**
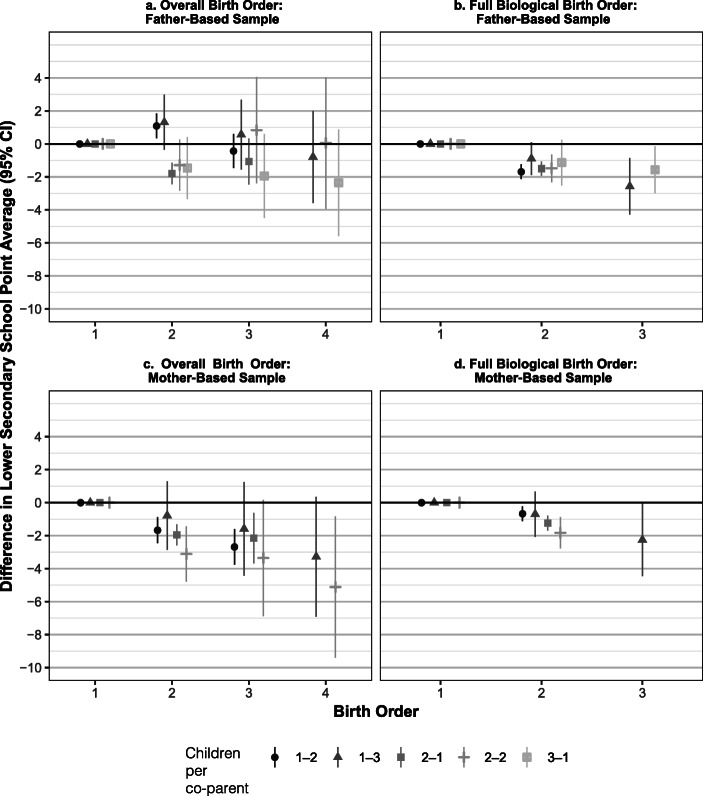
The association between school point average and the two types of birth order by different combinations of number of children per co-parent. The values for the different subgroups are indicated as follows: 1-2 indicates that a person has parented one child with the first co-parent, has parented two children with the second co-parent, and does not have any more children with a potential third co-parent; 2-2 indicates two children with each of two co-parents; and so on. We include analyses for subgroups with more than 800 observations, which is the number of observations needed to detect birth order effects in the nuclear family sample and explains why the 3-1 combination is included for only the father-based sample. The groups included constitute more than 90% of the full samples All models include controls for fathers’ and mothers’ age at birth, own cohort, and gender. The figure displays 95% confidence intervals. *Source*: The authors’ own calculations based on registry data.

Figure 1 shows the association between school points and birth order for each combination of the number of children per partner. Tables A1 and A2 of the online appendix provide the full numerical results for all birth order coefficients.^3^ Although the estimates are somewhat statistically imprecise because of small groups, there are some general patterns. There is a negative association between full biological birth order and school points, and the coefficients from each combination are similar. This finding holds across both samples and all combinations, as shown in panels b and d of Fig. 1.^4^ However, the association between overall birth order and school achievement differs between the two samples.

For children in the mother-based sample, having a higher overall birth order is negatively associated with educational achievement, as indicated in panel c of Fig. 1.^5^ However, the differences between the last sibling of the first set and the first child of the second set seem to be somewhat less marked in the 2-1 and 2-2 combinations. As previously discussed, a negative effect of union dissolution and related factors will typically affect all children of the first set negatively, which offsets at least some of the negative effects typically associated with having a higher birth order. For children born to mothers who display multipartnered fertility, the negative effects of birth order generally seem to trump these offsetting factors. Table A8 (online appendix) shows that this finding holds across the combinations for the full sample when the schooling achievements of the youngest sibling in the first set are compared with the oldest sibling in the second set and the variation in this difference is examined across the combinations of partners and children. The main finding is therefore a clear negative association between both types of birth order and educational achievement in the mother-based sample. In other words, all children achieve fewer school points on average compared with older half- *and* full siblings in this sample.

In the father-based sample, the general association between overall birth order and school point average is less clear, as seen in panel a of Fig. 1. In all combinations except 3-1, the oldest child of the second set tends to perform *better* in school when compared with the youngest sibling of the first set. This is most pronounced in the 1-2, 1-3, and 2-2 combinations, for which the coefficients for being second-born (1-2, 1-3) and third-born (2-2) are actually positive. Table A9 (online appendix) provides an estimate of this difference for the whole sample, and it generally holds across the combinations. Again, this is consistent with negative effects of union dissolution lowering the average achievement of the child/children of the first set. On the other hand, the associations between full biological birth order and educational achievement are consistently negative, as indicated in panel b of Fig. 1. Logically, this association can also be seen in panel a given that full biological birth order is a subset of overall birth order; later-born children *in the same set* tend to perform worse compared with their older full siblings. This finding implies that children in this sample typically perform on par with or better than the closest half-sibling but worse than full siblings.

**Fig. 2:**
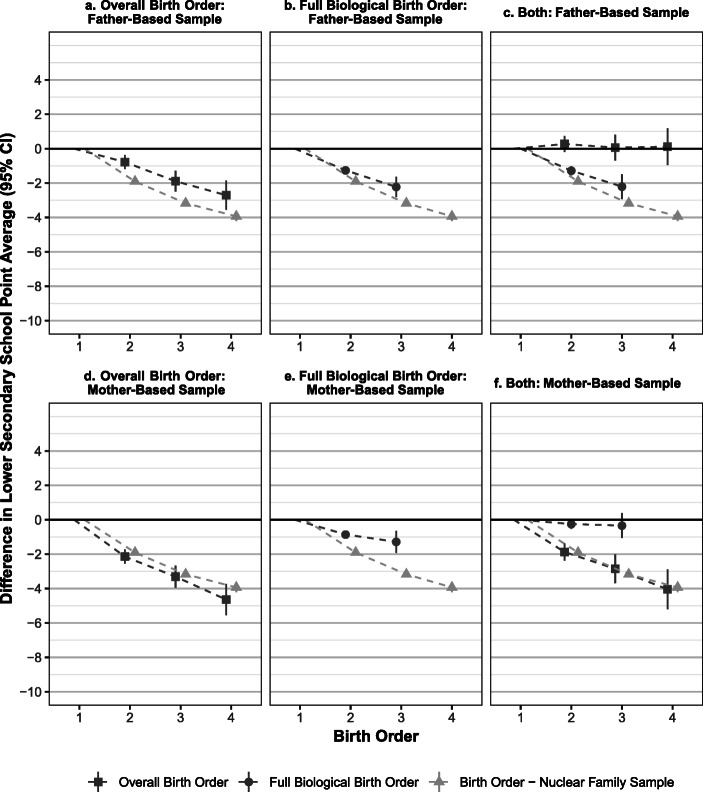
The differences in school point average at the end of compulsory education based on two types of birth order, with the firstborn^a^ as the reference in each case. Coefficients are shown for the two main samples as well as the nuclear family sample, where children with half-siblings are excluded (all panels). All coefficients indicate the difference in the average school points relative to the firstborn for the two types of birth order. Panels a–c display the results from Models 1–3 for the father-based sample. Panels d–f display the results from Models 1–3 for the mother-based sample. All results are based on fixed-effects regression within fathers/mothers (overall birth order) or their combination (full biological birth order). All models include controls for age of father at birth, age of mother at birth, and year of birth. The figure displays 95% confidence intervals. The reference groups for full biological and overall birth order are not identical, as specified in the variable definitions; they are overlapping only within the first set. *Source*: The authors’ own calculations based on registry data.

Figure 2 shows the associations between school achievement and overall and full biological birth order effects when all combinations of children per partner are pooled (with full results shown in Tables A3 and A4 in the online appendix). This enables us to investigate overall birth order patterns with higher statistical precision. The figure also includes results from the nuclear family sample for comparison (see Table A5 for full results).

Panels a, b, d, and e of Fig. 2 provide evidence for negative associations between the school point average and overall/full biological birth orders relative to older siblings with the same father (top row) and mother (bottom row). The association between school points and overall birth order is weaker in the father-based sample compared with that found in the nuclear family sample. This is consistent with the findings presented in panel a of Fig. 1, which indicate that negative effects of overall birth order cannot be clearly discerned for children with fathers displaying multipartnered fertility. The remaining negative association between *overall* birth order and school achievement in panel a of Fig. 2 is therefore likely to be driven by the negative association between school points and *full biological* birth order shown in panel b of Fig. 1. This is because of the substantial overlap between the full biological and overall birth order: overall birth order sequences are always composed of subsets of full biological birth orders. The question is therefore whether negative birth order effects operate mainly *across* or *within* sets of children depending on the sex of the parent displaying multipartnered fertility. Our third model takes both types of birth order into account simultaneously, thus providing additional evidence on this issue.

The results from Model 3 are presented in panels c and f of Fig. 2. Panel c indicates that when both types of birth order are included in the models, only the full biological birth order relative to siblings with the same *father* remains negatively associated with educational success. Per contra, the coefficient for the overall birth order is now small and not statistically significant. In the mother-based sample (panel f of Fig. 2), the opposite is true. Here, the overall birth order still matters, whereas the full biological birth order is no longer substantially or statistically significant. These arguably represent estimates of net effects of birth order in the context of multipartnered fertility given that we cannot isolate birth order effects from those of other factors, such as union dissolutions and family stability, using this model.

The negative associations between birth order and school achievement documented here are both statistically and substantially significant, and they are likely to have consequences for later school transitions. The largest disadvantages are found in the mother-based sample, where the difference between overall firstborns and second-borns is approximately 2 school points (roughly one-fifth of a standard deviation), whereas the difference between firstborns and fourth-borns is about 4 school points (roughly one-half of a standard deviation). The difference between first- and fourth-borns is of a magnitude similar to the sex gap in our sample.

## Discussion

### Summary of the Main Findings and Evaluation of Our Empirical Expectations

Previous studies on birth order and socioeconomic outcomes have typically studied nuclear families. However, recent demographic developments have led to increasingly complex family constellations. We acknowledge the importance of such developments by studying birth order effects on educational achievement in the context of multipartnered fertility. Based on the resource dilution and confluence theories as well as previous demographic research, we derive expectations for each type of birth order for each sample according to the sex of the parent who displayed multipartnered fertility.

We ask three research questions. Our first question asks whether associations between birth order and educational achievement exist in the context of multipartnered fertility. We provide evidence for overall negative associations between both full biological and overall birth order on the school point average at the end of compulsory schooling in both samples.

Our second and third research questions ask whether this association differs for full biological and overall birth order or according to the sex of the parent displaying multipartnered fertility. The answers to these two questions prove to be interrelated. In the mother-based sample, the association between overall and full biological birth order with educational achievement was negative. In the father-based sample, the overall birth order effect is weaker and less clear-cut, whereas the negative effect of full biological birth order remains important. Furthermore, when both overall and full biological birth order are included in the same model, only the overall birth order remains negatively associated with school achievements in the mother-based sample. For siblings in the father-based sample, this is the other way around, with only the full biological birth order remaining significant. We consider these associations as credible net effects of negative birth order effects due to the impact of union dissolution and family complexity, which clearly have a negative impact on school achievement.

We interpret these findings as indicating that the negative effects of birth order on educational achievement mainly operate *within* sets of children who share the same father and *across* sets for siblings born to the same mother. Asking whether one’s birth order relative to all siblings is more important for upper secondary school achievement than one’s birth order relative to full siblings yields a more intuitive interpretation. For siblings born to mothers displaying multipartnered fertility, the overall birth order across sets of siblings is clearly the most important: on average, these children perform worse in school compared with their older full and half-siblings, and the difference is similar in magnitude to that found in nuclear families.

For siblings born to fathers who display multipartnered fertility, the full biological birth order is what matters the most: these children tend to obtain fewer school points, on average, compared with older *full siblings* but also tend to perform equally well or better compared with the closest older *half-sibling*. The latter finding is likely due to factors other than typical birth order mechanisms, such as union dissolutions, family complexity, and differences in union stability. Although these mechanisms are relevant in both samples, the evidence indicates that they are not trumped by negative overall birth order effects in the mother sample. Still, there is clearly room for variation across the different subgroups, and future research should investigate this further.

Our findings are consistent with previous research. In a supplementary analysis, Kristensen and Bjerkedal (2010) considered birth order effects for mothers regardless of the number of co-parents and provided evidence for negative birth order effects on educational attainment at age 25. Other studies included control variables for union dissolution in the nuclear family birth order design, which did not make a difference (Kantarevic and Mechoulan 2006; Sigle-Rushton et al. 2014). However, the last two studies used two-parent fixed effects, and neither of the three took the issue of multipartnered fertility explicitly into account.

Our evidence supports the theoretical expectations that we derive from the resource dilution and confluence theories. In our expectations, we distinguish between the behavior of mothers and fathers who display multipartnered fertility. We argue that children who share a mother displaying multipartnered fertility will face a more consistent environment, where birth order effects can arise across sets. Fathers, on the other hand, are less likely to live with and interact with all their children at the same time for an extended period. This implies that although the father is often present in his children’s life for some time, we will not see the same consistency across sets in the father sample, thereby making full biological birth order effects more relevant. Based on the two theories, the question is whether the resource dilution and intellectual climate are mostly relevant only for full siblings in the father-based sample or both half- and full siblings in the mother-based sample. Our findings are consistent with these expectations. The confluence and resource dilution theories are therefore able to account for birth order patterns outside a nuclear family context. Still, our results indicate that they need modification or supplementation and need to take parental sex and related behavioral differences into account.

### Alternative Interpretations

Still, other theories and mechanisms could also provide relevant explanations. A more radical interpretation is that mechanisms specific to the mother are more important in explaining the birth order effects we observe, and possibly also birth order effects documented in other contexts.^6^ Several mechanisms are plausible based on the existing literature. For example, mothers in Western countries typically drink and smoke more during later pregnancies (Lundberg et al. 2010), and mothers breastfeed their first children more than their later-born children and are more likely to seek prenatal care for the oldest (Buckles and Kolka 2014). This interpretation also aligns with recent evidence on cognitive test scores from age 1 until school age using direct measures of maternal and paternal involvement. Only maternal involvement proved relevant in explaining negative birth order effects (Lehmann et al. 2018).

This mother-centric interpretation could favor the resource dilution theory, which can easily be modified to ascribe a greater importance to maternal resources and investments. In terms of the confluence theory, this interpretation would mean that the mother has a more positive influence on the average intellectual climate of the family compared with the father. This is hard to square with the fact that the confluence theory is based on the distinction between the intellectual levels of children and adults. Additionally, controlling for spacing does not affect our overall findings (see Tables A10 and A11 in the online appendix). Because spacing effects are emphasized in the confluence theory (e.g., Zajonc et al. 1979), this finding could indicate that mechanisms associated with the resource dilution theory are more important. Still, further data are necessary to separate the predictions of the two theories on a more fine-grained level, and evidence with direct measures of paternal and maternal quality time is mixed (Lehmann et al. 2018; Monfardini and See 2016).

It would be possible to adjudicate between our main explanation and the mother-centric interpretation if paternal investments could be measured directly given that the time spent with the father should not be driving birth order patterns if the first explanation is correct. However, as emphasized previously, we are unable to measure parental presence and investments nor the existence of shared residence arrangements directly. In a set of additional analyses with reduced samples, we included information on the number of years in which fathers lived with both the mother and their common children. We included information on years of cohabitation for each individual child as well as the average number of years in each set. This did not affect the main results appreciably (see Tables A12 and A13 in the online appendix). Still, this is a rough proxy for paternal involvement, and some fathers are more involved with nonresident children than others, while a very small group has full physical custody (Lyngstad et al. 2014).^7^

A third possibility, of course, is that other differences between the two samples are driving the findings. For example, perhaps the likelihood of the co-parents themselves displaying co-partner fertility could lead to differences between sets that are confounded with birth order mechanisms, for example, based on sex differences in the propensity for displaying this behavioral pattern. We investigated the relevance of this explanation by performing separate analyses depending on whether the first, second, or either co-parent themselves displayed multipartnered fertility (see Tables A14 and A15 in the online appendix). Despite differences in the magnitude of the estimates in the mother sample, the general pattern of full biological and overall birth order effects and mother/father sample remained: the differences were not statistically significant, and some subgroups were very small, thereby increasing statistical uncertainty.

### Sensitivity Checks and Remaining Limitations

Several analyses were also carried out to evaluate the robustness of our findings and assumptions. We performed two sensitivity analyses with slightly reduced samples in a further attempt to separate the negative consequences of union dissolutions from those of birth order.^8^ The first shows that accounting for union dissolutions does not affect the estimate for the nuclear family sample (Table A16, online appendix), indicating that the occurrence of union dissolutions does not affect our estimate of full biological birth order effects. In the second analysis, we considered only cases in which *both* unions of the parent displaying multipartnered fertility were dissolved in our two main samples, thus exposing both sets of children to this negative influence (Tables A17 and A18, online appendix). The findings were very similar to those of our main results, which indicates that the size of the downward bias in birth order effects caused by union dissolutions and related processes are not large enough to be detected given our sample size and statistical uncertainty in our main samples.

Our inclusion criteria require that parents have several children with multiple partners in a relatively short span of time (maximally 13 years between the first and the last), which could affect the external validity of our findings. However, previous research on multipartnered fertility has indicated that this fertility pacing is quite common for this group (Lappegård and Rønsen 2013). Also, previous studies have indicated that individuals displaying multipartnered fertility typically differ in their level of education from the rest of the population (Carlson and Furstenberg 2006; Evenhouse and Reilly 2016; Guzzo and Furstenberg 2007; Lappegård and Rønsen 2013; Thomson et al. 2014; Wiik and Dommermuth 2014). In four sensitivity analyses, looking at both our main samples and the nuclear family sample, we did not find that birth order effects vary in a statistically meaningful way based on either parent’s educational level (see Tables A19–A22, online appendix). This finding is in line with previous research on nuclear families (Black et al. 2005; Härkönen 2014). Therefore, we do not expect the magnitude of birth order effects documented in our study to be affected by the selection in our sample, at least not based on observable educational characteristics.

## Conclusion

We provide novel evidence for negative birth order effects that vary depending on the sex of the parent displaying multipartnered fertility. This type of fertility pattern has become increasingly common in western countries, and is likely to become even more common with time because of the continuation of relevant demographic trends and because of the likely transmission of the behavior across generations (Lappegård and Thomson 2018). Our main interpretation is that the pattern we document results from a combination of birth order mechanisms and residential arrangements. This makes our findings relevant for policy-makers given that the prevalence of different residential arrangements is influenced by both social norms and family policies (Cancian et al. 2014; Fehlberg et al. 2011; Sodermans et al. 2013). Shared residence between mothers and fathers has become more common in Norway and several other countries, and 50/50 shared residence has become a more important normative and political goal (Harris-Short 2010; Lyngstad et al. 2014; Singer 2008). This increase was particularly marked during the 2000s, whereas the youngest children in this study were born in 1998. During the 1980s and mid-1990s, shared residence was extremely rare in Norway (Jensen and Clausen 1997). Future studies should use data that include later cohorts or investigate countries with different family policies. Although such descriptive evidence is itself valuable, this could also enable adjudication between our suggestions that residence arrangements, the role of mothers more generally, or other potential mechanisms explain our birth order findings. Such research may provide new and important evidence to better understand the genesis of birth order effects, both in this context and more generally.

## Electronic supplementary material

ESM 1 (PDF 771 kb)

## Data Availability

The data required for this project were made available through the SEGREGATION project funded by the Research Council of Norway (RCN project #202479). These registry data are available from Statistics Norway to researchers with projects that satisfy the data owners’ requirements. For information on how to gain access to Norwegian microdata and formal requirements, see www.ssb.no/en/omssb/tjenester-og-verktoy/data-til-forskning.

## Footnotes

1 Birth order effects have been linked to *in utero* immunological responses and other biological processes (Dewey and Cohen 2007; Foster and Archer 1979). However, studies attempting to distinguish *in utero* influences from postnatal environments have found that the former are not important (Barclay 2015; Brenøe and Molitor 2015; Kristensen and Bjerkedal 2007).

2 Whenever we refer to differences or similarities between this sample and our main samples, such statements are based on pooling the samples and doing interaction tests (results are available upon request).

3 The slight differences between the estimates for full siblings and all siblings in certain overlapping combinations between the models in panels A and B of Table A1 on the one hand and panels A and B of Table A2 on the other is due to differences in the control variables when considering full siblings versus all siblings. The results are identical in a simple bivariate model. This does not affect our overall interpretations.

4 We also pooled the different combinations and estimated interactions between the birth order coefficients and each combination (results available upon request). The results are fully in line with those reported in Fig. 1 and panel B in both Table A1 and Table A2, with no statistically significant variation across the combinations.

5 An analysis with all combinations pooled and an interaction term between each combination and each birth order coefficient indicates no statistically detectable differences between the different combinations (results available upon request). This is in line with the results reported in Fig. 1 and panel A of Table A1.

6 We are here referring to the negative associations between birth order and educational achievements. To explain processes relating to union dissolutions, family stability, and so forth, other factors and the father’s role must clearly be taken into account, as previously discussed.

7 Although cohabitation data enable us to identify something close to fathers with sole custody, the number of individuals was too small for meaningful statistical analysis.

8 These samples include siblings in the 1987–1998 cohort. Information on cohabitation is available for this period only.
