# Supplementary material for: New Partner, New Order? Multipartnered Fertility and Birth Order Effects on Educational Achievement
Source: Demography. 2020 Sep 15;57(5):1625–46. doi: 10.1007/s13524-020-00905-4 (PMC7584560; doi:10.1007/s13524-020-00905-4)
Supplement: Supplementary file 1 — (PDF 771 kb) [file 13524_2020_905_MOESM1_ESM.pdf]

Online appendix

Section 1: Main Results

Table A1 Main results for different combinations of number of children and co-parents from mother-based samples: Estimated difference in lower–secondary school grade average for each higher overall birth order

	1-2	1-3	2-1	2-2
	(1)	(2)	(3)	(4)
A. Overall Birth Order (ref.= overall birth order 1)				
Overall birth order 2	–1.609***	–0.506	–2.018***	–3.206***
	(0.406)	(1.091)	(0.328)	(0.846)
Overall birth order 3	–2.743***	–1.331	–2.079**	–3.429
	(0.551)	(1.484)	(0.783)	(1.774)
Overall birth order 4		–3.046		–5.041*
		(1.895)		(2.164)
Constant	32.12***	31.55***	33.45***	35.95***
	(0.748)	(2.022)	(1.084)	(2.398)
Number of observations	9,276	1,173	6,339	1,176
B. Full Biological Birth Order (ref.= full biological birth order 1)				
Full biological birth order 2	–0.807***	–0.759	–1.316***	–1.820***
	(0.228)	(0.698)	(0.232)	(0.484)
Full biological birth order 3		–2.396*		
		(1.121)		
Constant	32.76***	31.72***	34.08***	36.66***
	(0.728)	(1.913)	(1.069)	(2.485)
Number of observations	9,276	1,173	6,339	1,176

Notes: Results in panel A are based on Model 1, which includes dummy variables for overall birth order; results in panel B are based on Model 2, which includes dummy variables for full biological birth order. All models include controls for fathers' and mothers' age at birth, own cohort, and gender. Standard errors are shown in parentheses.
Source: The authors' own calculations based on registry data.
*$p < .05$; **$p < .01$; ***$p < .001$

Table A2 Main results for different combinations of number of children and co-parents from father-based samples: Estimated difference in lower–secondary school grade average for each higher overall birth order (panel A) and each higher full biological birth order (panel B)

	1-2<br>(1)	1-3<br>(2)	2-1<br>(3)	2-2<br>(4)	3-1<br>(5)
A. Overall Birth Order (ref.= overall birth order 1)					
Overall birth order 2	1.127**	1.181	–1.853***	–1.567*	–2.241*
	(0.388)	(0.844)	(0.339)	(0.790)	(0.915)
Overall birth order 3	–0.424	0.303	–1.185	0.256	–2.737*
	(0.540)	(1.076)	(0.724)	(1.562)	(1.302)
Overall birth order 4		–1.111		–0.444	–4.039*
		(1.451)		(1.963)	(1.694)
Constant	32.64***	30.93***	32.91***	30.11***	33.09***
	(0.907)	(1.861)	(1.220)	(2.516)	(3.931)
Number of observations	9,576	1,990	5,736	1,452	842
B. Full Biological Birth Order (ref.= full biological birth order 1)					
Full biological birth order 2	–1.797***	–0.987	–1.630***	–1.549***	–1.178
	(0.233)	(0.503)	(0.227)	(0.427)	(0.713)
Full biological birth order 3		–2.689**			–1.830*
		(0.877)			(0.725)
Constant	31.76***	30.41***	32.65***	30.02***	33.21***
	(0.903)	(1.897)	(1.231)	(2.374)	(3.794)
Number of observations	9,573	1,990	5,736	1,452	842

Notes: Results in panel A are based on Model 1, which includes dummy variables for overall birth order; results in panel B are based on Model 2, which includes dummy variables for full biological birth order. All models include controls for fathers' and mothers' age at birth, own cohort, and gender. Standard errors are shown in parentheses.

Source: The authors' own calculations based on registry data.

$^*p < .05$; $^{**}p < .01$; $^{***}p < .001$

Table A3 Results for the full mother-based sample, coefficients from Models 1–3: Estimates. Estimated difference in lower–secondary school grade average for each higher birth order compared with the firstborn in each birth order type

	(1)<br>Overall	(2)<br>Full Biological	(3)<br>Both
Overall Birth Order (ref. = overall birth order 1)			
Overall birth order 2	–2.142***		–1.878***
	(0.212)		(0.257)
Overall birth order 3	–3.309***		–2.850***
	(0.323)		(0.424)
Overall birth order 4	–4.639***		–4.044***
	(0.465)		(0.589)
Full Biological Birth Order (ref. = full biological birth order 1)			
Full biological birth order 2		–0.858***	–0.250
		(0.108)	(0.140)
Full biological birth order 3		–1.284***	–0.339
		(0.328)	(0.367)
Constant	32.19***	33.07***	32.25***
	(0.537)	(0.524)	(0.538)
Number of Observations	19,508	19,508	19,508

Notes: Model 1 includes only dummy variables for overall birth order. Model 2 includes only dummy variables for the full biological birth order. Model 3 includes both types. All models include controls for fathers' and mothers' age at birth, own cohort, and gender. Standard errors are shown in parentheses

Source: The authors' own calculations based on registry data.

*$p < .05$; **$p < .01$; ***$p < .001$

Table A4 Results for the full father-based sample, coefficients from Models 1–3: Estimated difference in lower–secondary school grade average for each higher birth order compared with the firstborn in each birth order type

	(1)<br>Overall	(2)<br>Full Biological	(3)<br>Both
Overall Birth Order (ref. = 1)			
Overall birth order 2	–0.845***		0.223
	(0.213)		(0.243)
Overall birth order 3	–2.074***		–0.0848
	(0.316)		(0.390)
Overall birth order 4	–2.966***		–0.0998
	(0.442)		(0.559)
Full Biological Birth Order (ref. = 1)			
Full biological birth order 2		–1.311***	–1.301***
		(0.109)	(0.136)
Full biological birth order 3		–2.336***	–2.251***
		(0.301)	(0.369)
Constant	31.54***	31.57***	31.64***
	(0.621)	(0.611)	(0.622)
Number of Observations	20,776	20,776	20,776

Notes: Model 1 includes only dummy variables for overall birth order. Model 2 includes only dummy variables for the full biological birth order. Model 3 includes both types. All models include controls for fathers' and mothers' age at birth, own cohort, and gender. Standard errors are shown in parentheses

Source: The authors' own calculations based on registry data.

**p* < .05; ***p* < .01; ****p* < .001

Table A5 Results for the nuclear family sample: Estimated difference in lower–secondary school grade average by birth order compared with the firstborn

Birth Order	Coeff.
Birth Order 1 (ref.)	
Birth Order 2	–1.891***
	(0.0398)
Birth Order 3	–3.158***
	(0.0799)
Birth Order 4	–3.919***
	(0.139)
Constant	37.24***
	(0.259)
Number of Observations	376,088

Notes: Because there is no distinction between overall and full biological birth order in this sample, we refer to the coefficients as "birth order." The model was estimated with fixed effects for combinations of the two parents' IDs. All models include controls for fathers' and mothers' age at birth, own cohort, and gender. Standard errors are shown in parentheses

Source: The authors' own calculations based on registry data.

$*p < .05$; $**p < .01$; $***p < .001$

Section 2: Additional results

Table A6 Associations between birth order and school point average, including controls for the educational level of the co-parents. Mother-based sample

	(1)<br>Both + Controls	(2)<br>Both
Ref = Overall Birth Order 1		
Overall Birth Order 2	-1.657***	-1.681***
	(0.335)	(0.337)
Overall Birth Order 3	-2.437***	-2.574***
	(0.546)	(0.551)
Overall Birth Order 4	-3.732***	-4.023***
	(0.788)	(0.800)
Ref = Full Biological Birth Order 1		
Full Biological Birth Order 2	-0.250	-0.223
	(0.179)	(0.180)
Full Biological Birth Order 3	0.0691	0.175
	(0.495)	(0.497)
Controls for the educational level of co-parents	Yes	No
Constant	29.88***	33.41***
	(2.877)	(0.665)
Observations	19509	19509

Notes: The results of both columns are estimated based on model 3.The results presented in the first column includes controls for the educational level of all co-parents, measured in 2014.
All models include controls for the father`s and mother`s age at birth, own cohort and gender.
Source: The authors` own calculations based on registry data.
Standard errors in parentheses * $p < 0.05$, ** $p < 0.01$, *** $p < 0.001$

Table A7 Associations between birth order and school point average, including controls for the educational level of the co-parents. Father-based sample

	(1)<br>Both + Controls	(2)<br>Both
Ref = Overall Birth Order 1		
Overall Birth Order 2	0.272	0.223
	(0.239)	(0.243)
Overall Birth Order 3	0.0638	-0.0848
	(0.381)	(0.390)
Overall Birth Order 4	0.117	-0.0998
	(0.788)	(0.800)
Ref = Full Biological Birth Order 1		
Full Biological Birth Order 2	-1.280***	-1.301***
	(0.133)	(0.136)
Full Biological Birth Order 3	-2.203***	-2.251***
	(0.366)	(0.369)
Controls for the educational level of co-parents	No	Yes
Constant	26.70***	31.64***
	(1.903)	(0.622)
Observations	20776	20776

Notes: The results of both columns are estimated based on model 3.The results presented in the first column includes controls for the educational level of all co-parents, measured in 2014.
All models include controls for fathers` and mothers age at birth, own cohort and gender.
Source: The authors` own calculations based on registry data.
Standard errors in parentheses * $p < 0.05$, ** $p < 0.01$, *** $p < 0.001$

Table A8 Differences in school point average between the youngest sibling of the first set and the oldest sibling of the last set for each combination of partners and siblings. Mother-based sample

Oldest of second set (other combinations – ref.)	-0.951
	(0.565)
Oldest of second set #1-2	-0.784
	(0.571)
Oldest of second set #1-3	-0.237
	(0.795)
Oldest of second set #2-1	-0.0123
	(0.567)
Oldest of second set #2-2	1.144
	(0.781)
Constant	30.69***
	(0.785)
Observations	12303

Notes: The oldest of second set dummy has the value 0 for the youngest of the first set and 1 for the oldest of the second set. This dummy is interacted with each combination of children and partners to see whether the difference in school points between the youngest of the first and the oldest of the second varies for children between the different combinations. The combination variable includes four combinations (1-2, 1-3, 2-1,2-2, 3-1) with over 800 observations, with the rest of the combinations as the reference group.
All models include controls for fathers` and mothers age at birth, own cohort and gender.
Source: The authors` own calculations based on registry data.
Standard errors in parentheses * $p < 0.05$, ** $p < 0.01$, *** $p < 0.001$

Table A9 Differences in school point average between the youngest sibling of the first set and the oldest sibling of the last set for each combination of partners and siblings. Father-based sample

Oldest of second set (other combinations – ref.)	1.772** (0.676)
Oldest of second set #1-2	-0.534 (0.691)
Oldest of second set #1-3	-0.487 (0.813)
Oldest of second set #2-1	-0.550 (0.734)
Oldest of second set #2-2	0.268 (0.861)
Oldest of second set #3-1	-0.279 (0.988)
Constant	31.42*** (0.922)
Observations	12936

Notes: The oldest of second set dummy has the value 0 for the youngest of the first set and 1 for the oldest of the second set. This dummy is interacted with each combination of children and partners to see whether the difference in school points between the youngest of the first and the oldest of the second varies for children between the different combinations. The combination variable includes four combinations (1-2, 1-3, 2-1,2-2, 3-1) with over 800 observations, with the rest of the combinations as the reference group.
All models include controls for fathers` and mothers age at birth, own cohort and gender.
Source: The authors` own calculations based on registry data.
Standard errors in parentheses * $p < 0.05$, ** $p < 0.01$, *** $p < 0.001$

Table A10 Results from models 1-3 (full sample) including controls for spacing. Mother-based sample

	(1)<br>Overall	(2)<br>Overall + Controls	(3)<br>Full Biological	(4)<br>Full Biological + Controls	(5)<br>Both	(6)<br>Both + Controls
Ref. = Overall Birth Order 1						
Overall Birth Order 2	-2.142***	-2.138***			-1.878***	-1.836***
	(0.212)	(0.212)			(0.257)	(0.267)
Overall Birth Order 3	-3.309***	-3.211***			-2.850***	-2.888***
	(0.323)	(0.388)			(0.424)	(0.434)
Overall Birth Order 4	-4.639***	-4.480***			-4.044***	-4.142***
	(0.465)	(0.574)			(0.589)	(0.626)
Ref.= Full Biological Birth order 1						
Full Biological Birth order 2			-0.858***	-0.915***	-0.250	-0.297
			(0.108)	(0.130)	(0.140)	(0.165)
Full Biological Birth order 3			-1.284***	-1.365***	-0.339	-0.359
			(0.328)	(0.341)	(0.367)	(0.370)
Controls for spacing	No	Yes	No	Yes	No	Yes
Constant	32.19***	32.24***	33.07***	33.00***	32.25***	32.20***
	(0.537)	(0.548)	(0.524)	(0.533)	(0.538)	(0.550)
Observations	19508	19508	19508	19508	19508	19508

Notes: The spacing variable measures the years since the birth of the last-born older sibling, and is included as a set of dummy variables with zero (the value for the first born) as the reference category. Controlling for spacing of births generally does not seem to matter much in connection to birth order effects (see e.g. Black et al. 2005; Härkönen 2014). We still chose to include these variables here as conditioning on multipartnered fertility leaves us with a different sample compared to that of previous studies.
In this context spacing could be related to differential investment in different children based on their birth order for example or matter in other ways.
All models include controls for fathers` and mothers age at birth, own cohort and gender.
Source: The authors` own calculations based on registry data.
Standard errors in parentheses * $p < 0.05$, ** $p < 0.01$, *** $p < 0.001$

Table A11 Results from models 1-3 (full sample) including controls for spacing. Father-based sample

	(1)<br>Overall	(2)<br>Overall + Controls	(3)<br>Full Biological	(4)<br>Full Biological + Controls	(5)<br>Both	(6)<br>Both + Controls
Ref. = Overall Birth Order 1						
Overall Birth Order 2	-0.845***	-0.885***			0.223	0.220
	(0.213)	(0.212)			(0.243)	(0.249)
Overall Birth Order 3	-2.074***	-1.525***			-0.0848	-0.0825
	(0.316)	(0.353)			(0.390)	(0.398)
Overall Birth Order 4	-2.966***	-2.046***			-0.0998	-0.0939
	(0.442)	(0.509)			(0.559)	(0.585)
Ref.= Full Biological Birth order 1						
Full Biological Birth order 2			-1.311***	-1.228***	-1.301***	-1.298***
			(0.109)	(0.125)	(0.136)	(0.148)
Full Biological Birth order 3			-2.336***	-2.218***	-2.251***	-2.251***
			(0.301)	(0.311)	(0.369)	(0.369)
Controls for spacing	No	Yes	No	Yes	No	Yes
Constant	31.54***	31.88***	31.57***	31.69***	31.64***	31.65***
	(0.621)	(0.629)	(0.611)	(0.618)	(0.622)	(0.630)
Observations	20776	20776	20776	20776	20776	20776

Notes: The spacing variable measures the years since the birth of the last-born older sibling, and is included as a set of dummy variables with zero (the value for the first born) as the reference category. Controlling for spacing of births generally does not seem to matter much in connection to birth order effects (see e.g. Black et al. 2005; Härkönen 2014). We still chose to include these variables here, as conditioning on multipartnered fertility leaves us with a different sample compared to that of previous studies. In this context spacing could be related to differential investment in different children based on their birth order for example or matter in other ways.
All models include controls for fathers` and mothers age at birth, own cohort and gender.
Source: The authors` own calculations based on registry data.
Standard errors in parentheses * $p < 0.05$, ** $p < 0.01$, *** $p < 0.001$

Table A12 Full set of coefficients, with controls for the number of years cohabiting with both parents at the individual level. Reduced father-based sample

	(1)<br>Overall	(2)<br>Overall + Controls	(3)<br>Full Biological	(4)<br>Full Biological + Controls	(5)<br>Both	(6)<br>Both + Controls
Ref. = Overall Birth Order 1						
Overall Birth Order 2	-0.563*	-0.981***			0.411	-0.208
	(0.267)	(0.266)			(0.302)	(0.309)
Overall Birth Order 3	-1.601***	-1.864***			0.270	-0.434
	(0.397)	(0.394)			(0.485)	(0.491)
Overall Birth Order 4	-2.313***	-2.439***			0.424	-0.365
	(0.569)	(0.565)			(0.703)	(0.703)
Ref.= Full Biological Birth order 1						
Full Biological Birth order 2			-1.243***	-1.057***	-1.293***	-0.967***
			(0.134)	(0.137)	(0.166)	(0.173)
Full Biological Birth order 3			-2.149***	-1.699***	-2.228***	-1.694***
			(0.401)	(0.405)	(0.487)	(0.490)
Constant	26.49***	25.94***	26.65***	26.39***	26.83***	26.26***
	(2.182)	(2.112)	(2.176)	(2.098)	(2.178)	(2.105)
Observations	12959	12959	12959	12959	12959	12959

Notes: Based on reduced samples that include siblings in the 1987-1998 cohorts. Models 1-3 were estimated. The registries provides us with information on cohabitation for all individuals with common children, and we use this to construct a measure of the number of years (before age 16) that a child has lived with both parents or only the father, included as a continuous variable. All models include controls for fathers` and mothers age at birth, own cohort and gender.
Source: The authors` own calculations based on registry data
Standard errors in parentheses * $p < 0.05$, ** $p < 0.01$, *** $p < 0.001$

Table A13 Full set of birth order coefficients for different subgroups based on the average number of years cohabiting with both parents or only the father before age 16 on the sibling-level. Reduced father-based sample

	(1)<br>All	(2)<br>>5 Years	(3)<br><5 Years	(4)<br><2 Years
Ref.=Overall Birth Order 1				
Overall Birth Order 2	0.423	0.695	-0.129	-0.314
	(0.308)	(0.382)	(0.542)	(0.872)
Overall Birth Order 3	0.209	0.196	0.532	0.600
	(0.495)	(0.623)	(0.838)	(1.294)
Overall Birth Order 4	0.258	0.452	0.369	0.609
	(0.721)	(0.926)	(1.236)	(1.994)
Ref.=Full biological Birth Order 1				
Full biological Birth Order 2	-1.312^{***}	-1.284^{***}	-1.366^{***}	-1.471^{***}
	(0.169)	(0.215)	(0.300)	(0.529)
Full biological Birth Order 3	-2.255^{***}	-2.607^{***}	-1.581	-2.837
	(0.494)	(0.635)	(0.890)	(1.479)
Constant	31.41^{***}	31.68^{***}	32.79^{***}	31.47^{***}
	(0.740)	(0.949)	(1.225)	(2.061)
Observations	12959	8837	3830	1193

Notes: Based on reduced samples that include siblings in the 1987-1998 cohorts. The registries provide us with information on cohabitation for all individuals with common children, and we use this to construct a measure of the number of years (before age 16) that a child has lived with both parents or only the father. The variable we use to define the sub-sample in each column is based on the average of this measure, calculated for all children with the same father. All coefficients are reported for each subsample based on model 3, which includes both types of birth order simultaneously. All models include controls for fathers` and mothers age at birth, own cohort and gender.
Source: The authors` own calculations based on registry data.
Standard errors in parentheses * $p < 0.05$, ** $p < 0.01$, *** $p < 0.001$

Table A14 Results for all birth order coefficients for the full sample, as well as sub-samples where one or more co-parents also display multipartnered fertility. Mother-based samples

	(1)<br>All	(2)<br>First Co-parent Displays MPF	(3)<br>Second Co-parent Displays MPF	(4)<br>At least one Co-parent displays MPF
Ref.=Overall Birth Order 1				
Overall Birth Order 2	-1.936***	-1.428***	-4.495***	-2.088***
	(0.269)	(0.637)	(0.988)	(0.545)
Overall Birth Order 3	-2.922***	-2.158*	-6.513***	-2.954***
	(0.443)	(1.022)	(1.567)	(0.874)
Overall Birth Order 4	-4.170**	-3.239*	-8.519***	-4.517***
	(0.614)	(1.438)	(2.051)	(1.182)
Ref.=Full biological Birth Order 1				
Full biological Birth Order 2	-0.237	-0.746*	0.720	-0.351
	(0.145)	(0.322)	(0.569)	(0.286)
Full biological Birth Order 3	-0.304	-0.979	1.078	-0.186
	(0.370)	(0.893)	(1.215)	(0.698)
Constant	32.06***	31.14***	28.58***	30.63***
	(0.556)	(1.113)	(2.025)	(1.012)
Observations	19508	5939	2346	7803

Notes: All estimates based on model specification 3.
All models include controls for fathers` and mothers age at birth, own cohort and gender.
Source: The authors` own calculations based on registry data.
Standard errors in parentheses * $p < 0.05$, ** $p < 0.01$, *** $p < 0.001$

Table A15 Results for all birth order coefficients for the full sample, as well as sub-samples where one or more co-parents also display multipartnered fertility. Father-based samples

	(1)<br>All	(2)<br>First Co-parent Displays MPF	(3)<br>Second Co-parent Displays MPF	(4)<br>At least one Co-parent displays MPF
Ref.=Overall Birth Order 1				
Overall Birth Order 2	0.225	0.704	-1.102	-0.118
	(0.244)	(0.587)	(0.905)	(0.506)
Overall Birth Order 3	-0.0622	0.529	-1.501	-0.361
	(0.392)	(0.922)	(1.383)	(0.788)
Overall Birth Order 4	-0.0501	1.212	-1.327	0.0237
	(0.561)	(1.305)	(1.896)	(1.110)
Ref.=Full biological Birth Order 1				
Full biological Birth Order 2	-1.309***	-1.627***	-0.184	-1.177***
	(0.136)	(0.316)	(0.526)	(0.276)
Full biological Birth Order 3	-2.263***	-3.477***	-1.038	- 3.081***
	(0.369)	(0.928)	(1.731)	(0.837)
Constant	31.73***	31.82***	34.76***	32.18***
	(0.625)	(1.183)	(2.571)	(1.127)
Observations	20776	4040	1779	5503

Notes: All estimates based on model specification 3.
All models include controls for fathers` and mothers age at birth, own cohort and gender.
Source: The authors` own calculations based on registry data.
Standard errors in parentheses * $p < 0.05$, ** $p < 0.01$, *** $p < 0.001$

Table A16 Results from the nuclear family sample with and without a union dissolution taking place

	(1)<br>No Union Dissolution	(2)<br>Union dissolution Occurs	(3)<br>Union Dissolution Controls
Ref.= Birth Order 1			
Birth Order 2	-1.800***	-2.015***	-1.883***
	(0.0511)	(0.0926)	(0.0425)
Birth Order 3	-3.082***	-3.162***	-3.153***
	(0.104)	(0.191)	(0.0859)
Birth Order 4	-3.860***	-3.659***	-3.897***
	(0.174)	(0.361)	(0.148)
Constant	39.98***	35.47***	38.94***
	(0.692)	(0.425)	(0.401)
Observations	243614	102146	345760

Notes: These findings are based on a reduced version of the nuclear family sample that only includes the 1987-1998 cohorts, because of the need for information on cohabitation. In this sample, there is no distinction between the two types of birth order, so we simply refer to our measure as "Birth Order". The models were estimated as fixed effects analyses based on the mother`s and father`s identities. The second model only includes siblings from families where a union dissolution has taken place before the oldest child turned 15 (several thresholds were tested, and the findings were highly similar). In the third model controls for age at union dissolution are included for each separate sibling, with no union dissolution as the reference category.
All models include controls for fathers` and mothers age at birth, own cohort and gender.
Source: The authors` own calculations based on registry data.
Standard errors in parentheses * $p < 0.05$, ** $p < 0.01$, *** $p < 0.001$

Table A17 Results for models 1-3 with a union dissolution taking place in both sets. Reduced father-based sample.

	(1)<br>Overall	(2)<br>Specific	(3)<br>Both
Ref.= Overall Birth Order 1			
Overall Birth Order 2	-1.122**		-0.444
	(0.368)		(0.430)
Overall Birth Order 3	-1.816**		-0.659
	(0.568)		(0.699)
Overall Birth Order 4	-2.422**		-1.186
	(0.842)		(1.035)
Ref.= Full-biological Birth Order 1			
Full-biological Birth Order 2		-1.009***	-0.873***
		(0.191)	(0.237)
Constant	32.46***	32.61***	32.46***
	(0.980)	(0.971)	(0.982)
Observations	6568	6568	6568

Notes: These findings are based on a reduced version of the father-based sample that only includes the 1987-1998 cohorts, because of the need for information on cohabitation. The estimates are based on model specifications 1-3. The analyses include siblings born to mothers who experienced two union dissolutions taking place before the oldest child/children of both sets turned 15 (several thresholds were tested, and the findings were highly similar).
All models include controls for fathers` and mothers age at birth, own cohort and gender.
Source: The authors` own calculations based on registry data.
Standard errors in parentheses * $p < 0.05$, ** $p < 0.01$, *** $p < 0.001$

Table A18 Results for models 1-3 with a union dissolution taking place in both sets. Reduced mother-based sample.

	(1)<br>Overall	(2)<br>Specific	(3)<br>Both
Ref. = Overall Birth Order 1			
Overall Birth Order 2	-1.574***		-1.775***
	(0.396)		(0.487)
Overall Birth Order 3	-2.481***		-2.786***
	(0.608)		(0.796)
Overall Birth Order 4	-4.001***		-4.274***
	(0.986)		(1.217)
Ref.= Full-biological Birth Order 1			
Full-biological Birth Order 2		-0.372	0.202
		(0.204)	(0.263)
Constant	33.03***	33.65***	32.99***
	(0.889)	(0.868)	(0.888)
Observations	5801	5801	5801

Notes: These findings are based on a reduced version of the mother-based sample that only includes the 1987-1998 cohorts, because of the need for information on cohabitation. The estimates are based on model specifications 1-3. The analyses include siblings born to mothers who experienced two union dissolutions taking place before the oldest child/children of both sets turned 15 (several thresholds were tested, and the findings were highly similar).
All models include controls for fathers` and mothers age at birth, own cohort and gender.
Source: The authors` own calculations based on registry data.
Standard errors in parentheses * $p < 0.05$, ** $p < 0.01$, *** $p < 0.001$

Table A19 The associations between birth order and lower secondary school point average, by the educational level of the father. Nuclear family sample

	(1)<br>Lower	(2)<br>Secondary	(3)<br>Higher
Ref. = Birth Order 1			
Birth Order 2	-1.973***	-1.886***	-1.785***
	(0.0793)	(0.0625)	(0.0666)
Birth Order 3	-3.271***	-3.159***	-2.951***
	(0.158)	(0.126)	(0.134)
Birth Order 4	-3.863***	-3.998***	-3.666***
	(0.275)	(0.220)	(0.231)
Constant	33.10***	36.26***	43.41***
	(0.405)	(0.382)	(0.822)
Observations	102381	158096	114767

Notes: Since these results are based on the nuclear family sample, there is no distinction between the two types of birth order, so we simply refer to our measure as "Birth Order". The father`s level of education is measured in 2014. "Lower" refers to grades 1-10, i.e. elementary school and lower secondary school.
"Secondary" refers to grades 11-13, i.e. upper secondary school, and "Higher" to a bachelor degree or higher. All models include controls for the father`s and mother`s age at birth, own cohort and gender.
Source: The authors` calculations based on registry data.
Standard errors in parentheses * $p < 0.05$, ** $p < 0.01$, *** $p < 0.001$

Table A20 The associations between birth order and lower secondary school point average, by the educational level of the mother. Nuclear family sample

	(1)<br>Lower	(2)<br>Secondary	(3)<br>Higher
Ref. = Birth Order 1			
Birth Order 2	-1.951***	-2.034***	-1.614***
	(0.0769)	(0.0704)	(0.0602)
Birth Order 3	-3.269***	-3.345***	-2.638***
	(0.152)	(0.142)	(0.121)
Birth Order 4	-3.793***	-4.347***	-3.259***
	(0.264)	(0.257)	(0.204)
Constant	33.44***	35.77***	41.45***
	(0.389)	(0.448)	(0.594)
Observations	106067	121422	148170

Notes: Since these results are based on the nuclear family sample, there is no distinction between the two types of birth order, so we simply refer to our measure as "Birth Order". The mother`s level of education is measured in 2014. "Lower" refers to grades 1-10. "Secondary" refers to grades 11-13 and "Higher" to a bachelor degree or higher. All models include controls for the father`s and mother`s age at birth, own cohort and gender.
Source: The authors` calculations based on registry data.
Standard errors in parentheses * $p < 0.05$, ** $p < 0.01$, *** $p < 0.001$

Table A21 The associations between both types of birth order and lower secondary school point average, by the educational level of the father. Father-based main sample

	(1) Lower	(2) Secondary	(3) Higher
Ref. = Overall Birth Order 1			
Overall Birth Order 2	0.676	-0.122	0.189
	(0.381)	(0.391)	(0.552)
Overall Birth Order 3	0.449	-0.767	0.168
	(0.606)	(0.640)	(0.868)
Overall Birth Order 4	0.0272	-0.411	0.0164
	(0.870)	(0.911)	(1.238)
Ref. = Full Biological Birth Order 1			
Full Biological Birth Order 2	-1.639***	-0.932***	-1.438***
	(0.213)	(0.222)	(0.295)
Full Biological Birth Order 3	-1.760**	-2.983***	-1.792*
	(0.570)	(0.619)	(0.763)
Constant	29.97***	32.00***	33.79***
	(0.929)	(0.963)	(1.891)
Observations	8375	8252	3918

Notes: Each column display coefficients estimated based on model 3, with both types of birth order included.
The father`s level of education is measured in 2014.
“Lower” refers to grades 1-10, i.e. elementary school and lower secondary school.
“Secondary” refers to grades 11-13, i.e. upper secondary school, and “Higher” to a bachelor degree or higher. All models include controls for the father`s and mother`s age at birth, own cohort and gender.
Source: The authors` calculations based on registry data.
Standard errors in parentheses * $p < 0.05$, ** $p < 0.01$, *** $p < 0.001$

Table A22 The associations between both types of birth order and lower secondary school point average, by the educational level of the mother. Mother-based main sample

	(1)<br>Lower	(2)<br>Secondary	(3)<br>Higher
Ref. = Overall Birth Order 1			
Overall Birth Order 2	-1.975***	-1.378**	-2.115***
	(0.374)	(0.490)	(0.526)
Overall Birth Order 3	-2.986***	-1.950*	-3.626***
	(0.618)	(0.805)	(0.867)
Overall Birth Order 4	-4.426***	-2.446*	-5.180***
	(0.848)	(1.147)	(1.187)
Ref. = Full Biological Birth Order 1			
Full Biological Birth Order 2	-0.193	-0.573*	-0.0442
	(0.209)	(0.257)	(0.287)
Full Biological Birth Order 3	-0.658	-0.322	0.420
	(0.527)	(0.728)	(0.717)
Constant	30.21***	32.55***	35.96***
	(0.765)	(0.994)	(1.212)
Observations	8451	6094	4892

Notes: Each column display coefficients estimated based on model 3, with both types of birth order included. The mothers` level of education is measured in 2014.
Lower refers to grades 1-10. Secondary to grades 11-13 and higher to a bachelor degree or higher.
All models include controls for the father`s and mother`s age at birth, own cohort and gender.
Source: The authors` calculations based on registry data.
Standard errors in parentheses * $p < 0.05$, ** $p < 0.01$, *** $p < 0.001$
